# Pectoral nerve block in anesthesia for modified radical mastectomy: A meta-analysis based on randomized controlled trials

**DOI:** 10.1097/MD.0000000000015423

**Published:** 2019-05-03

**Authors:** Jia Zhao, Fanglei Han, Yang Yang, Hangyu Li, Zinan Li

**Affiliations:** a Department of Anesthesia, China-Japan Union Hospital of Jilin University; b Center for Applied Statistical Research and College of Mathematics, Jilin University, Changchun, China.

**Keywords:** general anesthesia, meta-analysis, modified radical mastectomy, pectoral nerve block

## Abstract

**Background:**

Many types of regional nerve blocks have been used during anesthesia for modified radical mastectomy. In recent years, the use of pectoral nerve (PECS) block has gained importance in postoperative analgesia, but there are still controversies regarding its efficacy. There is especially no consensus on the optimal type of PECS block to be used. Herein, we attempt to evaluate the analgesic efficacy of the PECS block after radical mastectomy.

**Methods:**

We searched PubMed, EMBASE, and the Cochrane library for randomized controlled trials (RCTs) for studies regarding PECS versus general anesthesia (GA) that were published prior to May 31, 2018. Outcome measures such as intra- and postoperative consumption of opioids, postoperative nausea and vomiting (PONV), need for postoperative rescue analgesia, and pain scores were analyzed. After quality evaluation and data extraction, a meta-analysis was performed using Review Manager 5.3 software, and the Grading of Recommendations Assessment, Development, and Evaluation (GRADE) system was used for rating the quality of evidence.

**Results:**

A total of 8 RCTs and 2 cohort studies involving 993 patients were eligible. Compared with the GA group, the PECS block group effectively reduced the intraoperative and postoperative use of opioid drugs, incidence of PONV, need for postoperative rescue analgesia, and pain scores within 0 to 6 hours after surgery. However, subgroup analysis showed that PECS I block did not have a significant advantage in reducing the intra- and postoperative consumption of opioids. Results for each outcome indicator were confirmed as having a high or moderate level of evidence.

**Conclusions:**

Even considering the limitations (evaluations of efficacy in different age groups and for chronic pain were not carried out) of this meta-analysis, it can be concluded that the PECS II block is an effective anesthetic regimen in modified radical mastectomy that can effectively reduce the intra- and postoperative consumption of opioids, postoperative PONV, and the need for postoperative rescue analgesia and can alleviate early pain (0–6 hours) after surgery.

## Abstract 2

Supplemental Digital Content is available in the text

## 1 Introduction

Breast cancer is the most common malignancy in women; surgery is one of the mainstays of treatment of breast cancer, and modified radical mastectomy is one of the standard treatments.^[1–3]^ Postoperative pain can seriously reduce the quality of life in patients, and acute pain can even trigger chronic pain syndrome. Thoracic paravertebral, thoracic epidural, intercostal nerve, and interscalene brachial plexus blocks have been used for anesthesia and abirritation during modified radical mastectomy, but their applications are limited by the complicated nature of the procedures and severe complications.^[1–4]^ In recent years, there has been increasing interest on a novel, less invasive nerve block, the pectoral nerve (PECS) block. PECS I block is an interfascial plane block administered between the pectoralis major and the pectoralis minor muscles. The PECS II block includes the PECS I block combined with a block administered above the serratus anterior muscle at the third rib.^[5,6]^ Numerous clinical trials have focused on the analgesic potential of the PECS block in breast augmentation surgery, small breast surgery, and breast cancer surgery, and have shown positive results. However, 2 recent studies indicated that the PECS block does not effectively block the sensory nerves nor does it exert additional analgesic effects. Therefore, there is some uncertainty about the clinical utility of the PECS block.^[7,8]^ We carried out this meta-analysis based on randomized controlled trials (RCTs) to elucidate the value of PECS in modified radical mastectomy with regard to the use of opioids, anesthetic complications, and analgesic effects. Moreover, the Grading of Recommendations Assessment, Development, and Evaluation (GRADE) pro system was used to verify the quality of evidence in order to provide reliable evidence for the clinical utility of the PECS block.

## 2 Methods

### 2.1 Search methods for identification of studies

This meta-analysis was conducted in accordance with the Preferred Reporting Items for Systematic Reviews and Meta-analyses (PRISMA) guidelines.^[9]^

The search strategy was designed according to the searching criteria issued by the Cochrane Collaboration. The search terms were a combination of MeSH terms and free terms, including “breast neoplasms, breast tumor, breast carcinoma; pectoral nerve, pectoral block, pectoral plane block; and general anesthesia.” Boolean operators were used to logically connect the search terms for literature retrieval in PubMed (1990–May 2018), EMBASE (1990–May 2018), and Cochrane (1990–May 2018) databases. In addition, we manually searched journals and reference lists for articles related to this study. The searching formula for PubMed was as follows: Search ((((((((((((((((((((((((((((((((Breast Neoplasm) OR Breast Tumors) OR Breast Tumor) OR Tumor, Breast) OR Tumors, Breast) OR Neoplasms, Breast) OR Breast Carcinoma) OR Breast Carcinomas) OR Carcinoma, Breast) OR Carcinomas, Breast) OR Mammary Neoplasms, Human) OR Human Mammary Neoplasm) OR Human Mammary Neoplasms) OR Neoplasm, Human Mammary) OR Neoplasms, Human Mammary) OR Mammary Neoplasm, Human) OR Breast Cancer) OR Cancer, Breast) OR Mammary Cancer) OR Cancer, Mammary) OR Cancers, Mammary) OR Mammary Cancers) OR Malignant Neoplasm of Breast) OR Breast Malignant Neoplasm) OR Breast Malignant Neoplasms) OR Malignant Tumor of Breast) OR Breast Malignant Tumor) OR Breast Malignant Tumors) OR Cancer of Breast) OR Cancer of the Breast)) AND (((General Anesthesias) OR General Anesthesia) OR Anesthesias, General)) AND ((((((((((((PECS) OR Pectoral nerve) OR Pectoral nerves) OR Pectoral block) OR Pectoral blocks) OR Pectoral I Block) OR Pectoral II Block) OR Pec blocks) OR Pec block) OR Pectoral plane blocks) OR Pectoral plane block) OR Pectoral plane). All analyses were based on previous published studies; thus, no ethical approval and patient consent are required.

### 2.2 Eligibility criteria

Eligible studies were required to meet all of the following criteria:

1. studies focusing on female patients with adult breast cancer who underwent modified radical mastectomy with no age, ethnicity, or nationality restrictions;
2. PECS block + general anesthesia (GA) as the experimental group, and pure GA (including placebo injection in the PECS block region) as the control group;
3. RCTs or high-quality cohort studies with no language limitations;
4. original literature with at least one of the following parameters: intraoperative opioid consumption, postoperative nausea and vomiting (PONV), pain scores (0, 6, and 24 hours after surgery), postoperative opioid consumption, and the number of patients requiring postoperative rescue analgesia; and
5. literature data that were true and credible and that could be converted into 2 categorical variables or continuous variables to represent the indicators.

Studies were considered ineligible and were excluded if they met the following criteria:

1. adequate preoperative physical assessment of the patient was not performed;
2. patients underwent secondary or non-radical surgery and breast reconstruction;
3. case reports, reviews, basic research on corpses, and conference papers without full text; and
4. it was not possible to extract valid data for the meta-analysis.

### 2.3 Outcome measurements

The outcome measures included:

1. intra- and postoperative consumption of opioids including sufentanil, fentanyl, and remifentanil, which are important for perioperative pain management and quality of recovery after surgery^[10–12]^;
2. incidence of PONV, which is a common complication of GA, and therefore, an important measure to evaluate the systemic response to anesthesia^[13,14]^;
3. pain scores at 0, 6, 12, and 24 hours postoperatively, which are helpful to evaluate the effects of the PECS block.^[15–17]^;
4. the number of patients requiring analgesic treatment with opioids such as fentanyl, morphine, and hydromorphone or non-opioids such as loxoprofen and acetaminophen within 24 hours after surgery (which is a common measure for evaluating the analgesic effect).

### 2.4 Assessment of methodological quality

Two reviewers (the first and second authors) independently assessed the quality of the included literature. The RCTs with scores <4 on the Jadad scale and cohort studies with scores <5 on the Newcastle-Ottawa Scale (NOS) indicated low quality.^[18]^ Divergences of opinion between the 2 reviewers were resolved by consulting a third reviewer (the corresponding author).

### 2.5 Data extraction

The 2 reviewers (the first and second authors) independently extracted data from all available studies in accordance with the standard form of data extraction. If disagreements occurred, the decision regarding data extraction was done by the third reviewer (the corresponding author).

For incomplete data, the reviewers tried to contact the authors of the original articles by email to request the original data, but did not receive a response. In some cases, the standard deviations (SD) that were not presented in the original reports could be estimated based on the range or median^[19]^ or based on the confidence interval (CI) as described in the Cochrane Handbook for Systematic Reviews of Interventions.^[20]^

### 2.6 Data analysis

Statistical analysis was performed using RevMan 5.3 software. The chi-square test was used to assess heterogeneity. If the value of *I*^2^ was > 50%, it was considered to indicate high heterogeneity, and a random effects model was used, otherwise a fixed effects model was used. Relative risk (RR) was used as the combined effect indicator for dichotomous variables; standardized mean differences were used for continuous variables.^[21]^ The 95% CI estimates and hypothesis test results for each variable were listed in a forest plot. For the outcome indicators with significant heterogeneity, a sensitivity analysis was conducted by removing the included studies one at a time to determine the sources of heterogeneity. Subgroup analysis was performed for each outcome indicator when these outcomes with regard to PECS I and PECS II blocks were reported in at least 2 articles each. A publication bias assessment using forest plots was intended to be conducted if no less than 10 studies were included. Finally, GRADE profile software was used to determine the level of evidence.

## 3 Results

### 3.1 Search results and characteristics of the selected studies

**Figure 1:**
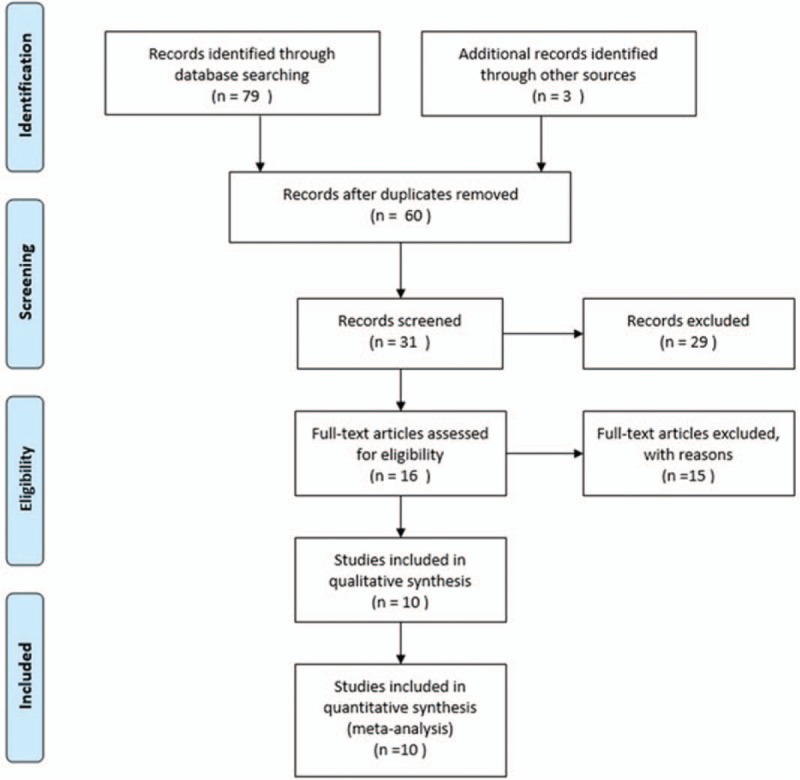
Flowchart of selection of studies.

Of the 82 potentially suitable studies, 8 RCTs and 2 cohort studies met our criteria; these included 2 studies that investigated PECS I, 7 that investigated PECS II, and 1 that investigated both PECS I and PECS II (Fig. 1).^[8,22–30]^ A total of 993 patients were enrolled in these 10 studies, 547 in the experimental group and 446 in the control group. The basic characteristics of the included studies are shown in Table 1. The Jadad scale and NOS scale were used for quality assessment of RCTs and cohort studies, respectively (Table 1). There were 8 high quality and 2 low quality studies. Six high-quality RCTs were double-blind studies.

**Table 1:**
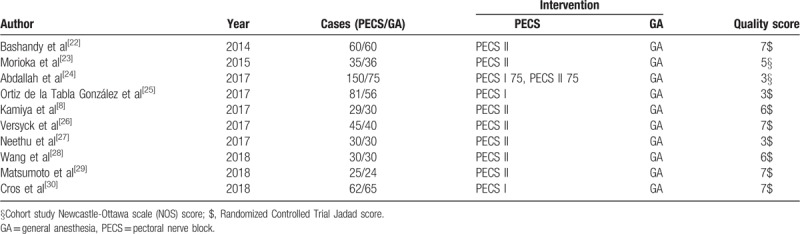
Main characteristics of all eligible studies included in the meta-analysis.

### 3.2 Comparison of intraoperative opioid consumption between the PECS group and the GA group

**Figure 2:**
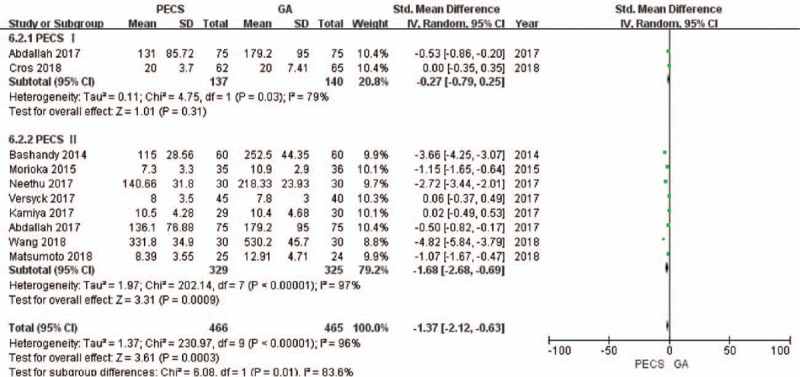
Forest plot of the patients’ intraoperative opioid consumption. PECS = pectoral nerve block, GA = general anesthesia.

A total of 9 studies that included 856 patients reported on the use of intraoperative opioids.^[8,22–24,26–30]^ Specifically, the study by Abdallah et al, evaluated both PECS I and PECS II.^[24]^ As *I*^2^ was 96%, the random effects model was used. Intraoperative opioid consumption in the experimental group was significantly lower than that in the control group (standardized mean difference [SMD] = −1.37, 95% CI [−2.12 to −0.63], *P* < .001]. No source of heterogeneity was found in the sensitivity analysis. In the subgroup analysis, there was no source of heterogeneity; moreover, there was no significant difference between the PECS I group and the control group (SMD −0.27, 95% CI [−0.79 to 0.25], *P* = .31]. The intraoperative opioid consumption in the PECS II group was significantly lower than that in the control group (SMD = −1.68, 95% CI [−2.68 to −0.69], *P* < .001] (Fig. 2).

The GRADE system showed a moderate level of evidence for the aforementioned results. (S1 Appendix)

### 3.3 Comparison of the incidence of PONV between the PECS group and the GA group

**Figure 3:**
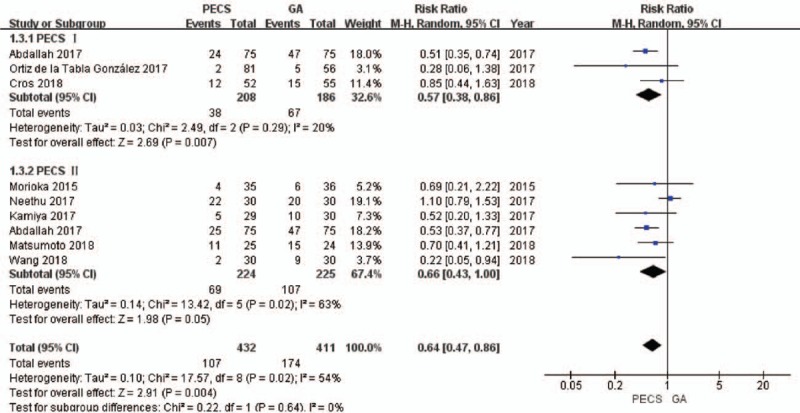
Forest plot of the incidence of postoperative nausea and vomiting in patients. PECS = pectoral nerve block, GA = general anesthesia.

A total of 8 studies^[8,23–25,27–30]^ that included 858 patients investigated PONV. As *I*^2^ was 54%, the random effects model was used. The incidence of PONV in the experimental group was significantly lower than that in the control group (RR = 0.64, 95% CI [0.47 to 0.86], *P* = .004]. The study by Neethu et al,^[27]^ was removed for sensitivity analysis, and the value of *I*^2^ was reduced to 0%. The fixed-effects model was then used, and the conclusion was unchanged (Fig. 3).

Results from the subgroup analysis showed that the PECS I and PECS II blocks effectively reduced the incidence of PONV [for PECS I, RR = 0.57, 95% CI [0.38 to 0.86], *P* = .007; for PECS II, RR = 0.66, 95% CI [0.43 to 1.00], *P* = .05].

The quality of the above evidence was ranked as high by the GRADE system. (S2 Appendix)

### 3.4 Comparison of pain scores 24 hours after surgery between the PECS group and the GA group

**Figure 4:**
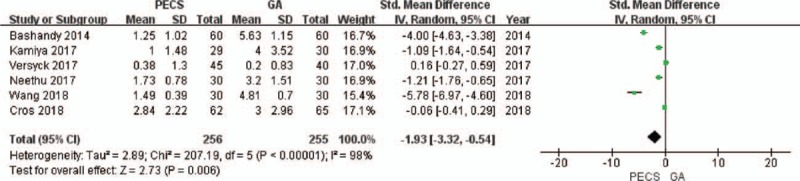
Forest plot of the patients’ pain scores (0 hour after surgery). GA = general anesthesia, PECS = pectoral nerve block.

**Figure 5:**
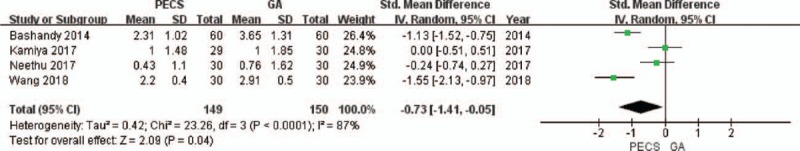
Forest plot of the patients’ pain scores (6 hours after surgery). GA = general anesthesia, PECS = pectoral nerve block.

**Figure 6:**
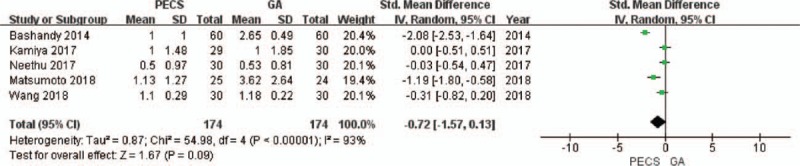
Forest plot of the patients’ pain scores (24 hours after surgery). GA = general anesthesia, PECS = pectoral nerve block.

A total of 6 studies^[8,22,26–28,30]^ that included 511 patients reported pain scores, of which, only the study by Neethu et al,^[27]^ reported the pain scores 12 hours after surgery. Therefore, it was impossible to conduct a relevant analysis on the pain scores (12 hours). The value of *I*^2^ was more than 50% in the data combination process, and the random effects model was used. At 0 (Fig. 4) and 6 hours (Fig. 5) after surgery, the pain scores of the experimental group were significantly lower than that of the control group (0 hour, SMD = −1.93, 95% CI [−3.32 to −0.54], *P* = .006; 6 hours, SMD = −0.73, 95% CI [−1.41 to −0.05], *P* = .04). At 24 hours after surgery (Fig. 6), there was no significant difference in the pain scores between the experimental group and the control group (SMD = −0.72, 95% CI [−1.57 to 0.13], *P* = .28). No source of heterogeneity was found in the sensitivity analysis.

The quality of the aforementioned evidence was evaluated as moderate by the GRADE system. (S3 Appendix)

### 3.5 Comparison of postoperative opioid consumption between the PECS group and the GA group

A total of 6 studies^[22,24,26–29]^ that included 725 patients reported the postoperative opioid consumption. The value of *I*^2^ was calculated to be 89%, and therefore, the random effects model was used. The postoperative opioid consumption in the experimental group was significantly lower than that in the control group (SMD = −1.15, 95% CI [−1.62 to −0.67], *P* < .001]. There was no heterogeneity in the sensitivity analysis.

**Figure 7:**
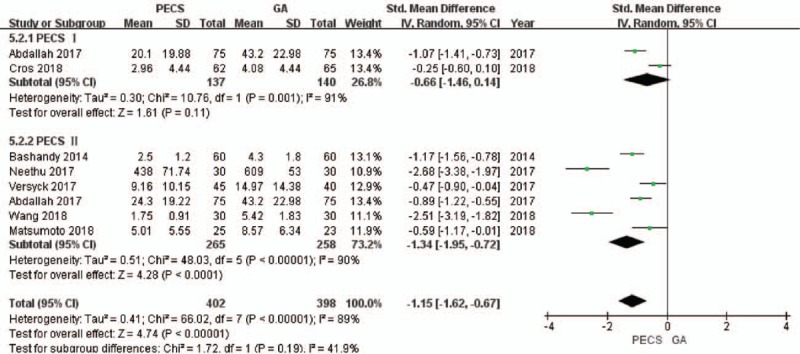
Forest plot of the patients’ postoperative opioid consumption. GA = general anesthesia, PECS = pectoral nerve block.

In the subgroup analysis, no source of heterogeneity was found. There was no significant difference between the PECS I group and the control group with regard to postoperative opioid consumption (SMD = −0.66, 95% CI [−1.46 to 0.14], *P* = .11], while the postoperative opioid consumption in the PECS II group was significantly lower than that in the control group (SMD = −1.34, 95% CI [−1.95 to −0.72], *P* < .001] (Fig. 7).

The GRADE system classified the level of the above evidence as moderate. (S4 Appendix)

### 3.6 Comparison of postoperative rescue analgesia between the PECS group and the GA group

A total of 6 studies^[8,22–26]^ that included 697 patients reported pain scores. The value of *I*^2^ = 0% indicated no heterogeneity, and the fixed-effects model was used. The number of patients requiring postoperative rescue analgesia in the experimental group was significantly lower than that in the control group (RR = 0.60, 95% CI [0.51 to 0.70], *P* < .001).

**Figure 8:**
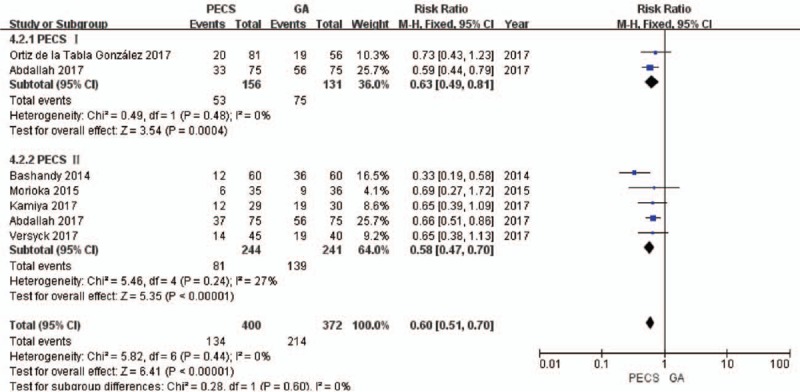
Forest plot of postoperative rescue analgesia needed by the patients. GA = general anesthesia, PECS = pectoral nerve block.

In the subgroup analysis, the number of patients requiring postoperative rescue analgesia in the PECS I and PECS II groups was significantly lower than that in the control group (for PECS I, RR = 0.63, 95% CI [0.49 to 0.81], *P* = .0004; for PECS II, RR = 0.58, 95% CI [0.47 to 0.70], *P* < .001) (Fig. 8).

The GRADE system ranked the level of the above evidence as high. (S5 Appendix)

## 4 Discussion

### 4.1 Key findings

This meta-analysis demonstrated that the PECS block, especially PECS II block, is a safe and effective option for analgesia in modified radical mastectomy. Compared with GA alone, PECS block combined with GA was more advantageous in reducing intraoperative opioid consumption, postoperative opioid consumption, postoperative early pain, incidence of PONV, and the need for postoperative rescue analgesia.

PECS block combined with GA can significantly reduce the amount of opioids used during the perioperative period. This is mainly due to the nerve block produced by the non-opioid drugs used in PECS block, which reduces the sensitivity of the nerve to intraoperative stimulation, alleviates muscle spasm, facilitates maintenance of the depth of anesthesia, and reduces the consumption of opioids during maintenance of anesthesia. The subgroup analysis indicated that PECS I block does not have the same advantages as PECS II block in reducing opioid use, as the pectoral nerve is a motor nerve containing only a small amount of sensory nerve fibers.^[31]^ Desroches et al^[7]^ found that PECS I block produced an effective motor blockade by anesthetic injection into the interfascial space between the pectoralis major and the pectoralis minor muscle, but could not produce a sensory block. The analgesic effects of PECS block are mainly dependent on the reduction of spasm after stimulation of the pectoralis major muscle. The PECS II block includes the PECS I block, and also entails blocking the intercostal nerve, thoracic nerve, and intercostal brachial nerve, which reduces the sensations on the skin of the thoracic wall and the armpit and achieves a greater range of analgesia.^[26]^ Although PECS I block by itself has limited analgesic effect and cannot effectively reduce the consumption of opioids, it can reduce postoperative chronic pain.^[7,31]^ In our meta-analysis, heterogeneity was not observed with regard to intraoperative and postoperative consumption of opioids. However, we believe that the differences in the choice, concentration, and dosage of the local anesthetic in each study may be the main source of heterogeneity. This may also be the reason for the level of evidence being evaluated as only moderate by the GRADE system.

The reduction in opioid use after PECS block combined with GA may have contributed to the lower incidence of PONV in these patients. PONV is a common side effect of opioid use. Cumulative opioid consumption after simple intravenous anesthesia and postoperative analgesia can cause itching, nausea and vomiting, gastrointestinal dysfunction, and intestinal obstruction, which results in some patients discontinuing the analgesic treatment or enduring the side effects. PECS block effectively reduces the incidence of PONV and improves the patient's quality of life. In the sensitivity analysis, the study by Neethu et al^[27]^ was the main source of heterogeneity, mainly because of the excessive proportion of overweight patients in the study (PECS group accounted for 46.67% of the patients and GA group accounted for 30% of the patients); overweight patients would require increased dosages of anesthetic and sedative drugs.

The results of our meta-analysis showed that early postoperative pain (0–6 hours) was significantly reduced in patients administered PECS block combined with GA as compared with those administered GA alone, but this difference gradually disappeared in the late postoperative period (24 hours). This is consistent with the initial reports.^[5,6]^ Postoperative pain can reduce the quality of life of patients. Controlling postoperative pain can help patients participate actively in postoperative rehabilitation and improve short-term and long-term recovery after surgery. It is the most important outcome measure of analgesic treatment. Visual Analogue Scale scores or Numerical Rating Scale scores are commonly used pain scores. Postoperative rescue analgesics, such as acetaminophen and diclofenac sodium, are often necessary when the original analgesic effect disappears. The need for postoperative rescue analgesia was lower when a combination of PECS block and GA was used, and there were marked advantages with the use of both PECS I and PECS II blocks in combination with GA. Not only does PECS II block cause motor blockade, but it also blocks the sensory nerves such as the thoracic nerve and the intercostal nerve, thereby effectively controlling the pain and reducing the need for postoperative analgesia. Although PECS I block could not effectively reduce the need for opioids, the lower need for postoperative analgesia affirms its analgesic effect in modified radical mastectomy. In addition, the thoracic nerve can transmit proprioceptive sensation and contains a small amount of pain nerve fibers, although it is described as a pure motor nerve in most textbooks.^[27]^

### 4.2 Strengths and limitations

This meta-analysis comprehensively evaluated a series of short-term indicators related to analgesic treatment, and especially focused on the pain scores at different time points (0, 6, 24 hours) after surgery as outcome indicators. The level of evidence was evaluated as moderate or high by the GRADE system, indicating a high credibility.

However, there are many deficiencies in this meta-analysis. There was a high heterogeneity in this meta-analysis. Since less than 10 studies were included, a funnel plotwas not conducted to find the sources of heterogeneity, and publication bias was not evaluated.

Some studies have shown that younger patients undergoing breast cancer surgery are more prone to develop persistent postoperative pain.^[8]^ However, based on the available data from the current studies, we could not evaluate the efficacy of the PECS block in patients of different ages and for chronic pain.

## 5 Conclusions

Based on the current research, we conclude that the PECS II block has obvious advantages with regard to all the outcome indicators and has definite analgesic effects, which is of value in modified radical mastectomy. Clinicians should further explore and optimize the analgesic treatment in patients, for example, addition of adrenaline to reduce the absorption of the local anesthetic during the nerve block.

## Author contributions

**Conceptualization:** Zinan Li.

**Data curation:** Jia Zhao.

**Formal analysis:** Jia Zhao, Hangyu Li.

**Investigation:** Fanglei Han.

**Methodology:** Jia Zhao.

**Resources:** Yang Yang.

**Software:** Fanglei Han.

**Supervision:** Zinan Li.

**Validation:** Yang Yang.

**Visualization:** Fanglei Han, Yang Yang, Hangyu Li.

**Writing – original draft:** Jia Zhao.

**Writing – review & editing:** Zinan Li.

## Supplementary Material

Supplemental Digital Content
