# Supplementary figures and images for: Pectoral nerve block in anesthesia for modified radical mastectomy: A meta-analysis based on randomized controlled trials
Source: Medicine (Baltimore). 2019 May 3;98(18):e15423. doi: 10.1097/MD.0000000000015423 (PMC6504333; doi:10.1097/MD.0000000000015423)

S1_appendix


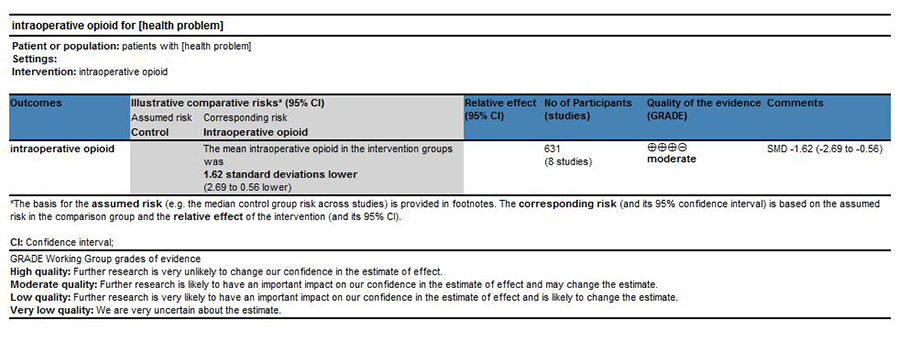


S2_appendix


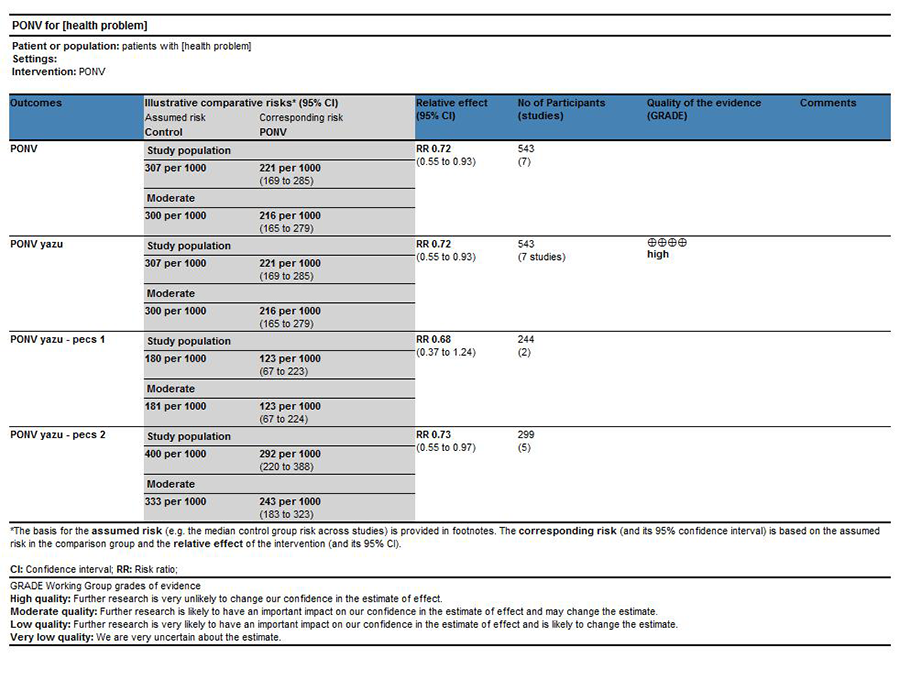


S3_appendix


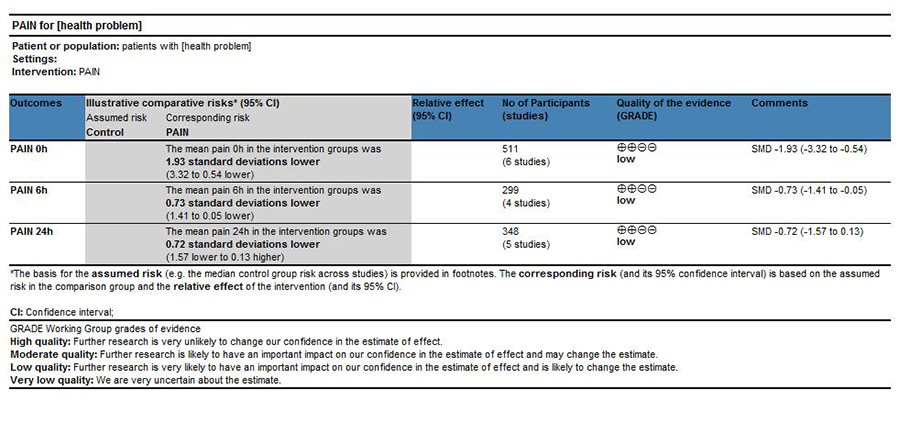


S4_appendix


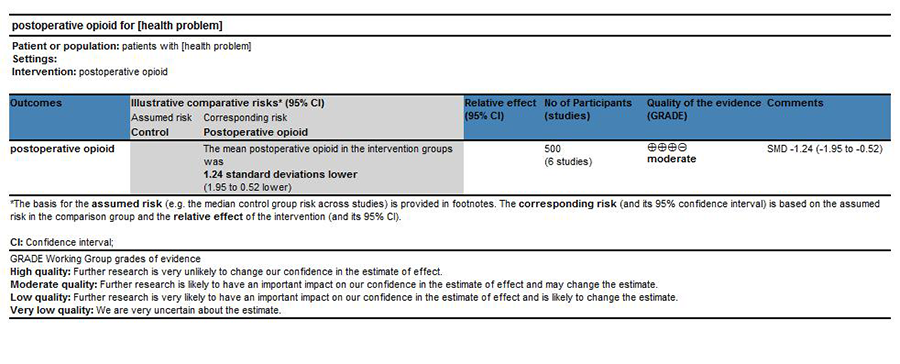


S5_appendix


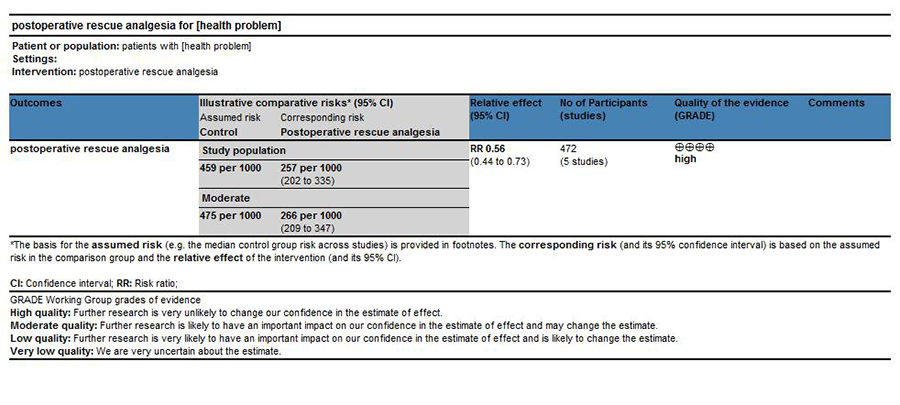

Supplement: Supplemental Digital Content [file medi-98-e15423-s001.doc]
